# Antioxidant and Anti-Apoptotic Properties of Oat Bran Protein Hydrolysates in Stressed Hepatic Cells

**DOI:** 10.3390/foods8050160

**Published:** 2019-05-11

**Authors:** Ramak Esfandi, William G. Willmore, Apollinaire Tsopmo

**Affiliations:** 1 Food Science and Nutrition Program, Department of Chemistry, Carleton University, 1125 Colonel By Drive, Ottawa, ON K1S 5B6, Canada; RamakEsfandi@cmail.carleton.ca; 2 Department of Biology, Carleton University, 1125 Colonel By Drive, Ottawa, ON K1S 5B6, Canada; Bill_Willmore@carleton.ca; 3 Institute of Biochemistry, Carleton University, 1125 Colonel By Drive, Ottawa, ON K1S 5B6, Canada

**Keywords:** food peptides, reactive oxygen species, antioxidant enzymes

## Abstract

The objective of this work was to find out how the method to extract proteins and subsequent enzymatic hydrolysis affect the ability of hepatic cells to resist oxidative stress. Proteins were isolated from oat brans in the presence of Cellulase (CPI) or Viscozyme (VPI). Four protein hydrolysates were produced from CPI and four others from VPI when they treated with Alcalase, Flavourzyme, Papain, or Protamex. Apart from CPI-Papain that reduced the viability of cell by 20%, no other hydrolysate was cytotoxic in the hepatic HepG2 cells. In the cytoprotection test, VPI-Papain and VPI-Flavourzyme fully prevented the damage due to peroxyl radical while CPI-Papain and CPI-Alcalase enhanced the cellular damage. Cells treated with VPI-hydrolysates reduced intracellular reactive oxygen species (ROS) by 20–40% and, also increased the intracellular concentration of glutathione, compared to CPI-hydrolysates. In antioxidant enzyme assays, although all hydrolysates enhanced the activity of both superoxide dismutase and catalase by up to 2- and 3.4-fold, respectively relative the control cells, the largest increase was due to VPI-Papain and VPI-Flavourzyme hydrolysates. In caspase-3 assays, hydrolysates with reduced ROS or enhanced antioxidant enzyme activities were able to reduce the activity of the pro-apoptotic enzyme, caspase-3 indicating that they prevented oxidative stress-induced cell death.

## 1. Introduction

The liver as a main detoxifying organ is prone to oxidative stress because of continuous exposure to reactive oxygen species (ROS) and toxicants. The production of ROS beyond the ability of cells to neutralize the radicals may cause damage to lipids, proteins and nucleotides which eventually can initiate liver-related injuries and diseases [1]. Cellular defence systems include small molecules and enzymes that are used to maintain a balance between oxidation and reduction. However, ageing, constant exposure to environmental toxicants or drugs can shift the balance towards a greater accumulation of oxidants (e.g., free radicals). It then becomes important to provide cells with additional amounts of antioxidants in the form of the supplement of formulated food products.

Antioxidant molecules have been considered as a strategy to prevent or reduce the incidence of many health-related conditions including liver diseases in which oxidative stress is present. Although there are synthetic antioxidants, natural ones have the advantage of being safer as well as being, in certain cases, multifunctional (e.g., antioxidant, anti-inflammatory, anti-apoptotic) [2,3,4]. Polyphenols have been extensively studied for their hepatic protective effect in animal and cell culture models, and this is summarized in a recent review [5]. The protection is often evaluated and quantified by electron donating compounds (e.g., vitamins E and C, glutathione) or antioxidant enzymes such as peroxidases and superoxide dismutase. In recent years, there has been an interest in the determination of the biological activity of hydrolyzed food proteins including hepato-protective effects. The hydrolyzed proteins contain peptides which are believed to be safer, and many are multifunctional [6]. In recent works, hydrolyzed corn proteins showed a hepatoprotective effect against carbon tetrachloride-induced liver injury in mice, characterized by a reduction in the oxidation of lipids, and an increase in the activity of superoxide dismutase and glutathione concentrations [7]. In hepatocyte cells, hydrolyzed rice bran proteins increased the intracellular glutathione concentrations by 2-fold in oxidatively stressed cells due to increased expression of γ-glutamylcysteine synthetase [8] while antioxidant peptides from microalgae had protection against alcohol-induced damage in HepG2/CYP2E1 cells [9]. 

Oat grains have the highest amount of proteins (up to 17% weight) amongst cereals most (50–80%) of which are globulins. Previous works on oat proteins resulted in the production of hydrolysates with radical scavenging activities [10,11], metal (calcium, iron) binding and inhibition of linoleic acid oxidation [12]. In one of those works showed that proteins extracted from oat brans with the aid of polysaccharide degrading enzymes, Cellulase and Viscozyme, had different susceptibility when hydrolyzed with Alcalase, Flavourzyme, Papain, and Protamex [13]. In addition, the extraction procedure also affected free radical scavenging and metal binding capacities of hydrolysates produced with the same protease. As a follow-up, this work aimed to determine at a cellular level, the potential of the hydrolysates to prevent oxidative stress and apoptosis in hepatic HepG2 cells.

## 2. Materials and Methods

### 2.1. Chemicals and Reagents

Caspase-3/CPP32 colorimetric assay Kit (Biovision, Catalog# K106-100) (# K106-100) was purchased from Biovision (Mountain View, CA, USA). Enzymes (glutathione peroxidase, catalase, copper-zinc superoxide dismutase, xanthine oxidase, glutathione reductase (GR)), 3-(4,5-dimethylthiazol-2-yl)-2,5-diphenyltetrazolium bromide (MTT), reduced glutathione (GSH), oxidized glutathione (GSSG), nicotinamide adenine dinucleotide phosphate (NADPH), 4-(2-hydroxyethyl)-1-piperazineethanesulfonic acid (HEPES), dihydrochlorofluorescein diacetate (DCFH_2_-DA), bathocuproinedisulfonic acid disodium salt (BCS), nitroblue tetrazolium chloride (NBT), diethylenetriaminepentaacetic acid (DETAPAC), xanthine, 5,5-dithiobis-(2-nitrobenzoic acid) (DTNB), triethanolamine, 2-vinylpyridine were obtained from Sigma Aldrich (Oakville, ON, Canada). Hydrogen peroxide (H_2_O_2_), 2,2′-azobis(2-amidinopropane) dihydrochloride (AAPH), sodium azide (NaN_3_), ethylenediaminetetraacetic acid disodium (EDTA), antibiotic-antimycotic (100×) were obtained from Fisher Scientific (Ottawa, ON, Canada).

### 2.2. Oat Bran Protein Hydrolysates

Oat protein isolates and their hydrolysates used in this study are from recent work [13]. Briefly, polysaccharides in defatted brans were cleaved (pH 4.5, 45 °C, 90 min) with either Cellulase (20 units/g) or Viscozyme (3 units/g). The Cellulase Protein Isolate (CPI) and the Viscozyme Protein Isolate (VPI) were subsequently obtained after solubilisation at pH 9.5 and precipitation at isoelectric point (pH 4.5). Each protein isolate was digested with proteolytic enzymes (Alcalase, Flavourzyme, Papain and Protamex), to produce eight protein hydrolysates. The CPI-derived hydrolysates were named CPI-Al, CPI-Fl, CPI-Pa, and CPI-Pr, respectively while the ones from VPI were named VPI-Al, VPI-Fl, VPI-Pa, and VPI-Pr, respectively. Detailed preparation methods and protein contents are as provided in the literature [13].

### 2.3. Cell Culture, Cytotoxicity and Cytoprotective Experiments

Human hepatic carcinoma HepG2 cells (ATCC^®^ HB-8065™) were obtained from Cedarlane Laboratories Ltd (Burlington, ON, Canada). They were grown in Dulbecco’s Modified Eagle Media (DMEM) with 10% fetal bovine serum (FBS, from Wisent Bioproducts St-Bruno, QC, Canada). Cells were passaged every 3–4 days and maintained in a humidified incubator at 37 °C supplemented with 5% CO_2_-95% air. 

The cytotoxicity and cytoprotection effects of protein hydrolysates were evaluated using a modified MTT assay described by Nair and Liu, 2010 [14]. The concentration of protein hydrolysates was 100 µg/mL as previous work on other hydrolysates found no toxic effect up to 1 mg/mL [15]. A volume of 200 µL cells at a density of 2 × 10^4^ cells/mL (in DMEM containing 10% FBS and 1% antibiotic) was transferred to clear 96-well tissue culture plates and incubated for 24 h. Media was then removed, and the cells washed with phosphate buffer saline solution (PBS, pH 7.2) followed by 24 h incubation with protein hydrolysates and another wash. Subsequently, 200 µL of media was added to the cells intended for cytotoxicity studies while 200 µL of media containing 10 mM of AAPH were added to those used for cytoprotection evaluation. After another 24 h incubation, cells were washed twice with PBS. The determination of viable cells in each case was initiated by adding 100 µL of media and 50 µL MTT (1 mg/mL) were added to all wells and maintained for 1 h at 37 °C. The MTT solution was replaced with 50 µL of DMSO before absorbance reading (570 nm) with background subtraction (630 nm). The cytotoxicity effect of proteins hydrolysates was compared to normal cells (i.e., no treatment), the cytoprotection was to both normal, and AAPH treated cells.

### 2.4. Determination of Intracellular Reactive Oxygen Species

Reactive oxygen species (ROS) scavenging activities of protein hydrolysates were determined based on a reported procedure [16]. Briefly, HepG2 Cells were seeded at 4 × 10^4^ cell/mL (200 µL/well) in a dark 96-well tissue culture plate and incubated for 24 h prior after which they were washed with PBS (2 × 200 µL, pH 7.4). Media with or without protein hydrolysates were added to the wells and allowed to interact for 24 h. Cells were washed again with PBS followed by addition of AAPH (10 mM), interaction over 45 min and another wash. To determine ROS, 200 µL of 40 mM DCFH_2_-DA in 10 mM HEPES buffer was added to the wells. Fluorescent intensity was immediately measured over 60 min with 5 min intervals for one hour, using Fluostar Optima, (BMG Labtech, Offenburg, Germany) at an excitation wavelength at 485 nm and emission at 530 nm. Untreated cells were used as the negative control (NEG), whereas the positive control (POS) consists of cells treated with AAPH only. ROS was calculated according to the equation below and normalized to protein contents from the Lowry assay.

ROS (%) = (((Final reading − Initial reading)/Initial reading) × 100)/(mg protein/mL).
(1)

### 2.5. Preparation of Cell Extracts for Glutathione and Enzyme Assays

HepG2 cells were seeded at 5 × 10^5^ cell/plate in 60 mm tissue culture plates and incubated at 37 °C for 24 h after which media was discarded, and the plates washed with PBS (2 × 4 mL, pH 7.4). Hydrolyzed proteins (4 mL in media) were added to plates and incubated for 24 h. Cells were washed with PBS and treated with 10 mM AAPH (4 mL in media) for 24 h. Cells were detached with 0.25% trypsin (0.5 mL) over 5 min at 37 °C, inhibited with 1 mL culture media, transferred to 1.5 mL vials. The suspension was centrifuged at 1000× *g* for 5 min at 4 °C. Cells were collected and re-suspended in 1 mL ice-cold potassium phosphate buffer (0.1 M, pH 7.5) containing 5 mM EDTA, 0.1% Triton X, and 0.6% sulfosalicylic acid to be lysed by sonication for determination of antioxidant enzyme activities or in the lysis buffer provided in the Caspase-3 assay kit. The lysis on ice used a probe-type sonicator (Vibra-Cell, Sonics & Materials Inc., Newtown, CT, USA) with cycles set at 1 min for 15 s on and 10 s off. For glutathione assay, 1 mL of 5% ice-cold sulfosalicylic acid bubbled with 100% nitrogen was used for cell lysis. Cell extracts (supernatants) were obtained by centrifugation at 13,000× *g* for 5 min and immediately used for assays.

### 2.6. Glutathione Assay

Total and oxidized glutathione were measured based on a method described by Rahman, et al. [17]. Total glutathione measurement was done by adding to a 96-well clear microplate, 20 µL of cell extracts, GSH standard (0.103–26.4 µM), or buffer (for blank) and an equal volume (60 µL) of 0.8 mM DTNB and 100 IU/mL of glutathione reductase. The reaction rate between GSH in the lysate and DTNB were recorded at 412 every 20 s for 2 min after the addition of 60 µL of 0.8 mM β-NADPH. In the case of oxidized glutathione, 2 µL of 2-vinylpyridine (0.185 mM) were added to 100 µL of cell extract, GSSG standard (0.103–26.4 µM), or blank in 1.5 mL microcentrifuge tubes, mixed and incubated for 1 h at room temperature. Then, 6 µL of 6-times diluted triethanolamine were added to the tubes and vortex mixed. After 10 min incubation time, the next steps were as above for total glutathione. Concentrations were calculated based on a standard curve of glutathione normalized to protein content.

### 2.7. Antioxidant Enzyme Assays

#### 2.7.1. Catalase

Catalase activity was measured using a method described by Beers and Sizer [18]. Cell extracts 200 µL) were mixed with 1790 µL of potassium phosphate buffer (0.05 M, pH 7.0) in UV disposable cuvettes. Ten microliters of 30% H_2_O_2_ were added followed by measurement of its decomposition are at 240 nm using a Cary 50 Bio UV-Vis spectrophotometer with 18-cell changer (Varian Inc., Mississauga, ON, Canada). The rate was used to calculate catalase activity and expressed as a percentage of the control.

#### 2.7.2. Glutathione Peroxidase

The activity of glutathione peroxidase (GPx) was measured based on previous literature [19]. A potassium phosphate stock buffer (0.05 M, pH 7.0) containing 1.1 mM EDTA and 1.1 mM NaN_3_ was used to make to GSH (1 mM), and 1 unit/mL glutathione reductase (1 IU/mL) before the assay and NADPH (4 mM) that was kept on ice. Into a clear 96 well-plate, 187.5 µL of the above buffer was added followed by 12.5 µL of NADPH and 25 µL of either cell lysate or GPx standards (0.02–0.1 U/mL) or buffer for the blank. The reaction was initiated by the addition of 2.5 mM H_2_O_2_ and incubated at 30 °C for 5 min then, the change in the absorbance was measured at 340 nm due to the disappearance of NADPH over 4 min was used to calculate the activity as Units of GPx activity/mg protein. 

#### 2.7.3. Superoxide Dismutase

The activity of superoxide dismutase was measured using a procedure described by Spitz and Oberley with modifications [20]. The assay solution was 50 mM phosphate buffer (pH 7.8) containing DETAPAC (1.25 mM), BSA (0.16 mg/mL), Catalase (1.25 U/mL), NBT (70 µM), xanthine (125 mM), and BCS (62.5 µM). Xanthine oxidase 0.06 U/mL was made in 1.34 mM DETAPAC buffer. Cell extracts were diluted to eight concentrations (2–500 mg/mL) while CuZnSOD standard concentrations were 2–500 ng/mL. Specifically, 200 µL of the assay solution and 25 µL of sample or standard were added to a clear 96-well microplate. The reaction then initiated by the addition of 25 µL xanthine oxidase followed by kinetic measurement every 15 s for 3 min and at 560 nm. Rates of reduced of NBT were used to determine concentrations of cell lysate that inhibited 50% of the activity of SOD enzyme.

### 2.8. Caspase-3 Assay

Caspase-3/CPP32 Colorimetric Assay Kit (Biovision, Catalog #K 106-100) was used to determine the apoptosis induced by AAPH and with or without pre-treatment of cells with protein hydrolysates. The analysis was done according to the manufacturer’s instructions. In summary, cell lysates were diluted to obtain 50 μg protein/mL. Then, 50 μL of diluted lysate was mixed with 50 μL of reaction buffer containing 10 mM DTT and 5 μL of 4 mM Asp-Glu-Val-Asp *p*-nitroanilide (DEVD-pNA) substrate. Tubes were incubated at 37 °C for 2 h followed by measurement of the absorbance at 405 nm. A blank was also prepared using cell lysis buffer only.

### 2.9. Statistical Analysis

All experiments were performed in triplicate (*n* = 3), and one-way analysis of variance (ANOVA) tests (SAS^®^ Software, SAS OnDemand, 9.4, 2017, SAS Institute Inc., Cary, NC, USA) were used to compare mean values. Significant differences were evaluated using Duncan’s multiple range test (*p* < 0.05). 

## 3. Results and Discussion

### 3.1. Cytotoxicity and Cytoprotection

**Figure 1:**
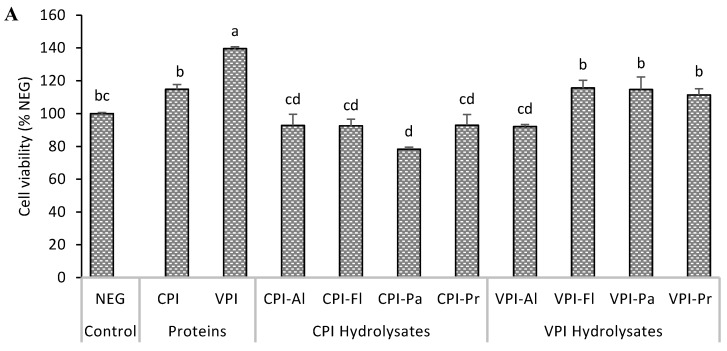

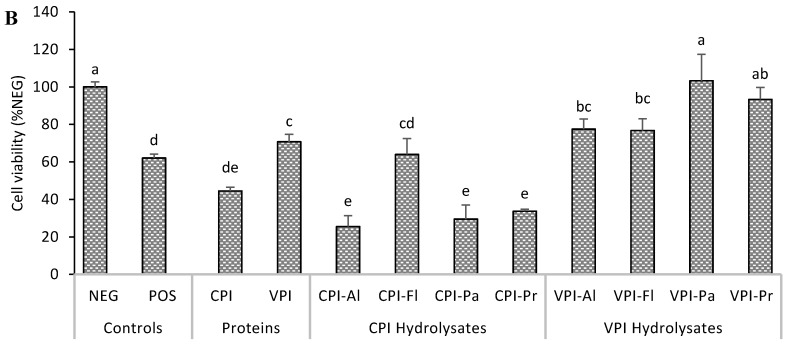
Cytotoxicity (**A**) and cytoprotective (**B**) effects of oat bran protein hydrolysates, digested with Cellulase (CPI) or with Viscozyme (VPI) and their hydrolysates produced with Alcalase (Al), Flavourzyme (Fl), Papain (Pa) or Protamex (Pr) in HepG2 cells. Cells were exposed to hydrolysate (100 µg/mL) for 24 h (**A**) or exposed to hydrolysates for 24 h followed by AAPH for 24 h (**A**) before the determination of viable cells. NEG: normal cells; POS: cells treated with AAPH alone. Data with different letters showed significant difference (*p* < 0.05) from Duncan’s multiple range test (*n* = 3).

The reduction of the MTT tetrazolium salt to formazan, by mitochondrial oxidoreductase enzymes, was used to determine the cytotoxicity and cytoprotection of hydrolyzed oat proteins. The concentration of formazan is proportional to the number of viable cells. In the cytotoxicity test (Figure 1A), exposure of HepG2 cells to protein hydrolysates showed that only CPI-Pa significantly reduced the number of viable cells. CPI-Pa reduction was about 21%. Four hydrolysates reduce the viability by about 7% (*p* < 0.05) while three others increased the viability by 11–15 % (*p* > 0.05). One of the non-hydrolyzed proteins, CPI, did not affect cell growth while the other, VPI, significantly enhance the growth by about 40%. Cell growth promotion has been reported using food protein hydrolysates. In one study, for example, soy protein hydrolysate enhanced the growth of mouse hybridoma ME-750 cells by 7% while the increase was 48% for wheat gluten hydrolysate [21]. The effect of protein hydrolysates then appeared to vary depending on the source of proteins, the protein extraction procedure and the specificity of the proteases used. Similar to CPI-Pa, an Alcalase gelatin hydrolysate was reported to decrease the viability of HepG2 cells by 20% at 200 µg/mL [22]. The beneficial effect of hydrolyzed proteins of cells may come from their nutritive or regulatory effects but also, potentially from their action on maintaining the redox balance. 

The exposure of HepG2 cells to AAPH radical caused a significant decrease in the viability of cells (Figure 1B), and this is consistent with previous works on oxidatively-induced cellular damage [23,24]. AAPH induced oxidative stress reduced cell viability to 62.1% ± 2.1% relative to normal control cells. When the cells were pre-treated with hydrolysate before being stressed, different effects were found. VPI-Pa and VPI-Pr caused no reduction in viability, relative to normal cells, and was, therefore, the most cytoprotective. The other protective hydrolysates were VPI-Al and VPI-Fl. CPI-FL did not affect the oxidant activity (i.e., AAPH) while the remaining three hydrolysates caused a decrease in viability (Figure 1B).

It appeared that hydrolysates from Viscozyme extracted proteins (VPI) had better cytoprotection than those derived from Cellulase extracted proteins (CPI). Previous work found that the composition and the concentration of polypeptides in CPI and VPI were different and this translated in different peptides composition hydrolysates [13]. That work identified 92, 171 and 609 peptides made of 8–26 amino acids in VPI-Pa, CPI-Pa, and VPI-Pr, respectively. The activity of the hydrolysate is related to several factors that may include the number of peptides, their size, charge or hydrophobicity and the overall sequences. 

### 3.2. Determination of Intracellular Reactive Species and Glutathione

**Figure 2:**
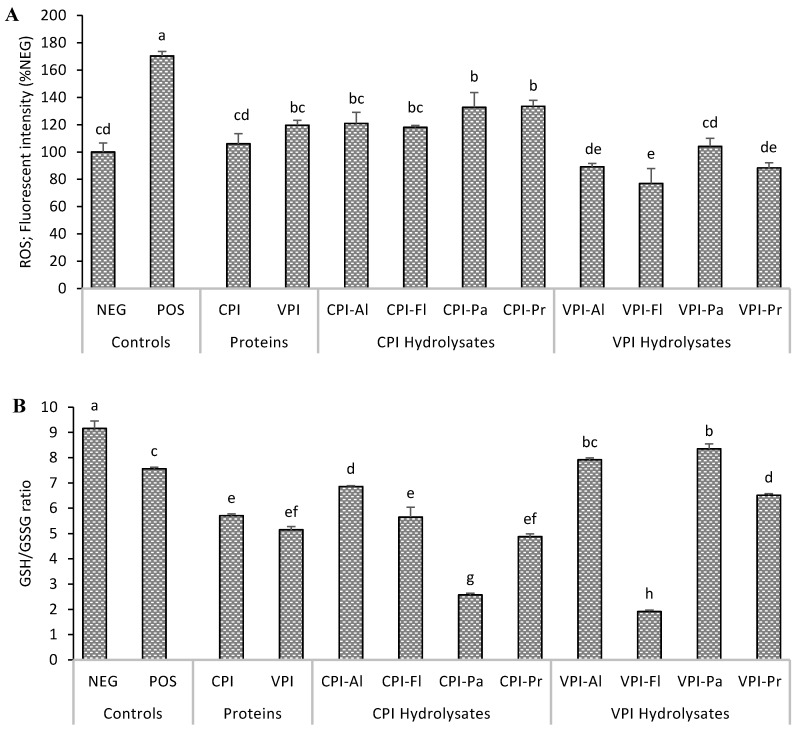
Intracellular reactive oxygen species (ROS) (**A**) and GSH/GSSG ratio (**B**). HepG2 cells were treated with oat bran proteins extracted with Cellulase (CPI), Viscozyme (VPI) and their Alcalase (Al), Flavourzyme (Fl), Papain (Pa) or Protamex (Pr) hydrolysates (100 µg/mL) for 24 h, followed by exposure to AAPH for 24 h, before obtaining the data. NEG: normal cells; POS: cells treated with AAPH alone. Data with different letters showed significant difference (*p* < 0.05) from Duncan’s multiple range test (*n* = 3).

The scavenging activities of the protein hydrolysates against AAPH-generated intracellular oxidants are displayed in Figure 2A. It is known that when DCFH_2_-DA diffuses through the cell membrane, it is de-acetylated into DCFH_2_ inside the cytosol which is then oxidized by intracellular ROS to form fluorescent DCF [23]. As expected, treatment of HepG2 cells with AAPH significantly increased the production of intracellular ROS to 170.4% ± 3.2% relative to the NEG control. All oat bran protein hydrolysates significantly reduced ROS relative to the minus AAPH NEG control. The most significant reduction 77–104% of control normal cells was associated with pre-treatment with VPI hydrolysates, and this correlated well with their cytoprotection data (i.e. greater viability of cells). This is likely because peptides in VPI hydrolysates, or their metabolites, were transported more easily inside the cells. Similar to this work, hydrolyzed proteins from Nile tilapia were found to reduced ROS in HepG2 cells [24]; meanwhile, no effect was observed for bean globulin hydrolysates on Caco-2 cells [25]. The effect hydrolyzed proteins on intracellular ROS can then vary based on the source of proteins but also on the cellular model. 

The oxidative status of the HepG2 cells was further evaluated by measuring the concentration of glutathione, an important cellular redox molecule. In the presence of ROS, GSH is oxidized to GSSG which can be converted back to its reduced form by glutathione reductase [26]. The ratio of GSH to GSSG is then a reliable representation of the cellular redox status. A greater ratio corresponds to less oxidative stress. In this work, HepG2 cells stressed with AAPH had a 24.0% lower ratio GSH/GSSG ratio compared to normal NEG cells (Figure 2B), indicating partial depletion of reduced GSH which might translate into an inability to maintain the redox balance. However, when HepG2 cells were pre-treated with hydrolysates, VPI-Al and VPI-Pa resulted in a higher ratio of GSH/GSSG than the AAPH NEG control group (without AAPH). In the presence of other hydrolysates, the cellular environment was more oxidized as GSH/GSSG ratios were similar or lower compared to AAPH control. Cells pre-treated with VPI-Al and VPI-Pa, and which have a higher ratio of GSH/GSSH, are amongst those showed less intracellular ROS and greater viability. This likely gives these cells the greatest ability to maintain redox balance.

In our previous work, tested the radical scavenging activities of hydrolysates used in the present study and determined the sequences of peptides in CPI-Pa, VPI-Pa, and VPI-Pr using tandem mass spectrometry [13]. It was found that CPI-Pa and VPI-Pa had similar scavenging power for peroxyl radicals; meanwhile, VPI-Pa had higher superoxide anion radical scavenging and ferrous iron chelating activities. The reason was attributed to higher ratios of charged residues and histidine in VPI-Pa relative to CPI-Pa but also its higher content of peptides with less than 10 residues (42% in VPI-Pa and 37% in CPI-Pa). Smaller peptides in VPI-Pa might then explained its better protection in most assays (e.g., ROS, GSH, cytoprotection) against AAPH-induced cellular injury. This is likely because smaller peptides are less degraded by cellular proteases. 

### 3.3. Antioxidant Enzymes Activity

**Figure 3:**
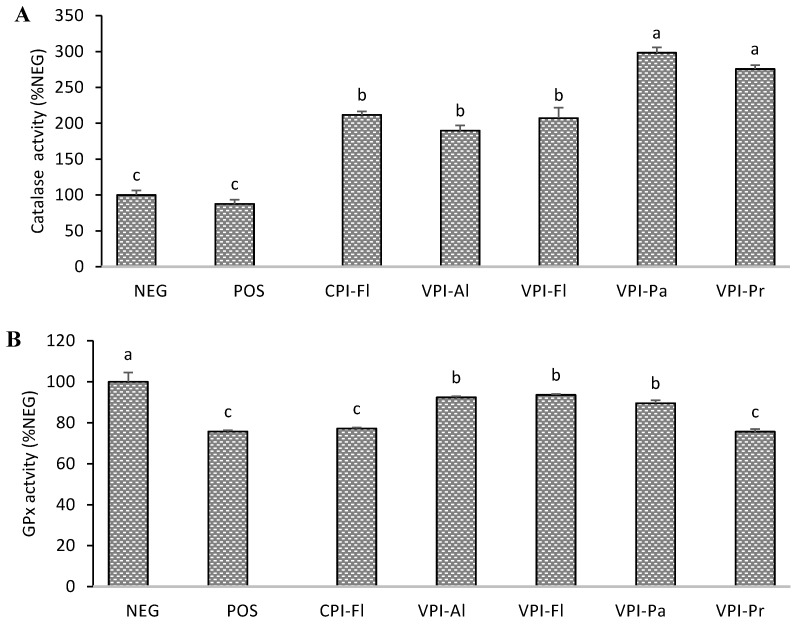

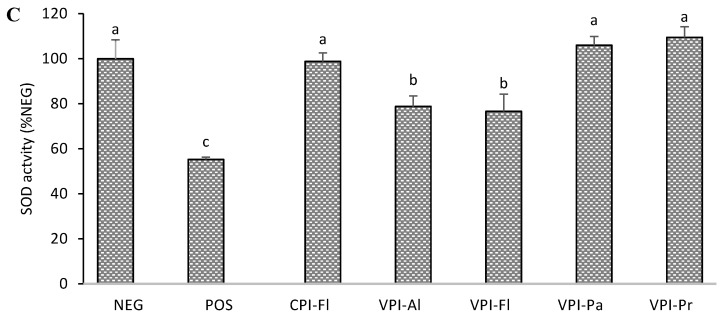
Activity of the antioxidant enzymes in AAPH-stressed HepG2 cells of hydrolysates (100 µg/mL) that showed lower reactive oxygen species (ROS) and higher GSH/GSSG ratios. Catalase (**A**); glutathione peroxidase (**B**); superoxide dismutase (**C**). NEG: normal cells (no treatment); POS: positive control (AAPH treated only). Oat bran proteins extracted with Cellulase (CPI) or with Viscozyme (VPI) and then hydrolyzed with Alcalase (Al), Flavourzyme (Fl), Papain (Pa) or Protamex (Pr). Data with different letters showed significant difference (*p* < 0.05) from Duncan’s multiple range test (*n* = 3).

The enzymes catalase, glutathione peroxidase (GPx) and superoxide dismutase (SOD) are important in the prevention of oxidative stress through their action on ROS which are converted into stable or less reactive species. The hydrolysates, CPI-Fl, VPI-Al, VPI-Fl, VPI-Pa, and VPI-Pr were selected for this section because they were either cytoprotective, produced less ROS, or had a higher amount of GSH. The induction of oxidative stress by AAPH reduced the activity of catalase, glutathione peroxidase (GPx), and superoxide dismutase (SOD) to 87.5% ± 6.1%, 75.7% ± 0.7% and 55.2% ± 1.1%, respectively in relation to normal cells (Figure 3). The change was, however, not significant in the case of catalase. The activity of catalase increased by up to 3-fold when cells were pre-treated either of the five hydrolysates with VPI-Pa having the most up-regulation (Figure 3A). A similar increase was reported in HepG2 due to the action of peptide GLVYIL [23].

Figure 3B showed partial recovery of GPx activity in HepG2 when they were pre-treated with three (VPI-Al, VPI-Fl and VPI-Pa) of the five hydrolysates (Figure 3B). There was no change in GPx activities of CPI-Fl and VPI-Pr. GPx is important for the conversion of hydrogen peroxide and hydroperoxides, which are reactive molecules, into the water and hydroxylated molecules. The activity of GPx is also dependent on GSH supply and is therefore not surprising that the hydrolysates with higher activities are also the ones with greater GSH contents. Other studies have also reported a positive effect of food protein hydrolysates (e.g. rice, fish) on the activity of GPx in hepatocyte cells [27,28].

In the SOD assay, three of the hydrolysates (CPI-FL, VPI-Pa, and VPI-Pr) completely prevented the adverse effects of AAPH-induced oxidative stress of the enzyme activity (Figure 3C). The two others (VPI-Fl, VPI-AL) partially prevented the loss of SOD activities but, this was still significant. Casein hydrolysates had an enhancing effect on SOD activities in lymphocyte cells [29] while a similar effect was observed for fish gelatin hydrolysates in human colon cells [22].

Catalase is more resistant to inactivation by peroxyl radicals due to the narrowness of its active site which can explain why it was the least affected by AAPH. It has however been reported that lipid peroxides might cause induction of the catalase [30] which explained the 3-fold increase found in this work. SOD converts superoxide anion radical to hydrogen peroxide which is subsequently converted to water by catalase. The two hydrolysates that produced the highest SOD activities also led to highest catalase activities, and this is a relevant presumption as proper elimination of hydrogen peroxide is important to prevent the loss of SOD activity. It is possible that peptides present in oat protein hydrolysates may have induced the expression of catalase and SOD genes as reported in a recent work [31].

The difference in composition of peptides and their size might explain their behaviour on the cellular antioxidant enzymes. In the previous work, larger peptides in VPI-Pr contained 26 residues compared to 20 in VPI-Pa while 31% of peptides in VPI-Pr were less than 10 residues relative to 42% in VPI-Pa which was due to higher proteolysis activity of Papain [13]. The enzymes tested in this work are metalloenzymes, peptides in hydrolysates that interfere in metal (iron, selenium, zinc or manganese) update or metabolism can then affect the activity of catalase, GPx or SOD. For example, it has been reported that iron uptake by cells was stimulated by radicals [32] and this could explain the increased of catalase activity we observed in the presence of AAPH. It was also found that the decrease in GPx activity in the liver was associated in impairment of selenium absorption [33]. Peptides in hydrolyzed oat proteins might have prevented the oxidation of the selenocysteine at the active site of GPx.

### 3.4. Caspase-3 Determination and Cell Apoptosis

Caspase-3 enzyme belongs to a family of proteases that exists in an inactive form, and its activation is highly regulated. These proteases are responsible for programmed cell death, control cell disintegration and prevent the release of their content into the intracellular matrix which will damage the surrounding cells [34]. Caspase-3, one of the executioner caspases, is important specifically since its presence is mostly an indicator of irreversible cell death [34]. This enzyme can cleave Asp-Glu-Val-Asp *p*-nitroanilide (DEVD-pNA) and release *p*-nitroaniline (pNA). The absorbance of pNA is proportional to the enzyme activity, and hence, higher intensities indicate more apoptosis.

**Figure 4:**
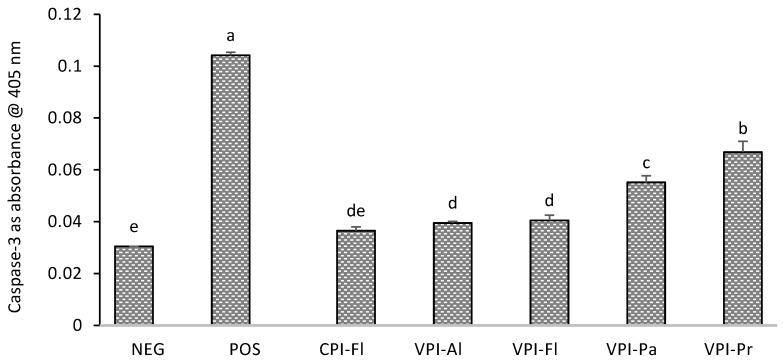
Changes in absorbance corresponding to the change in the activity of caspase-3. HepG2 were hydrolysates (100 µg/mL) for 24 followed by exposure to AAPH for 24 h before determining the activity using a Kit. Oat bran proteins extracted with Cellulase (CPI) or with Viscozyme (VPI) and then hydrolyzed with Alcalase (Al), Flavourzyme (Fl), Papain (Pa) or Protamex (Pr). NEG: normal cells (no treatment); POS: positive control (treated with AAPH only). Data with different letters showed significant difference (*p* < 0.05) from Duncan’s multiple range test (*n* = 3).

Treatment of HepG2 cells with AAPH increased the activity of caspase-3 by approximately 3-fold (Figure 4), which translated in greater proteolytic activity and therefore greater cells death. Pre-treatment of cells with the hydrolysates resulted in a reduction of 2- to 3-fold in caspase-3 activity, relative to the AAPH NEG control. Only one the hydrolysates, CPI-Fl, restored caspase-3 activity to the level found in the non-treated group. There are different mechanisms proposed as for how the antioxidant protein hydrolysates can exert their effect. Some of the hydrolysates with higher cell viability and greater catalase and SOD activities did not possess lower caspase-3 activities, likely because there are different anti-apoptotic mechanisms. Whey protein hydrolysates, for example, showed an anti-apoptotic effect in PC12 cells by up-regulating the expression of anti-apoptotic Bcl-2, a regulatory protein that can proteolytically cleave pro-apoptotic proteins and reduce the expression of the pro-apoptotic Bax protein [35]. In endothelial cells, the anti-apoptotic properties of seahorse protein hydrolysates down-regulated caspase-3 and p53 and increased the Bcl-2/Bax ratio [36]. Another potential mechanism is the upregulation of poly (ADP-ribose) polymerase which is responsible for DNA repair [37]. In addition to caspase-3, some of the above mechanisms might have been involved in the protection provided by the hydrolyzed oat bran proteins. Peptides in hydrolyzed oat proteins might then have protected HepG2 cells through some of these mechanisms rather than through caspase-3 inhibition. 

## 4. Conclusions

Oat bran protein isolates, and their enzymatic digests, protected hepatic HepG2 cells from induced oxidative stress. The protection of protein isolated was dependent on the extraction procedure, as well as whether Cellulase or Viscozyme was used. The protection of hydrolysates was related to the extraction procedure and the protease utilized. Hydrolysates derived from Viscozyme extracted proteins had the highest antioxidant activities; specifically, those of Papain and Flavouzyme. Future work will investigate the activity of peptides present in these hydrolysates.
